# Determination of Polypeptide Antibiotic Residues in Food of Animal Origin by Ultra-High-Performance Liquid Chromatography-Tandem Mass Spectrometry

**DOI:** 10.3390/molecules25143261

**Published:** 2020-07-17

**Authors:** Tomasz Bladek, Iwona Szymanek-Bany, Andrzej Posyniak

**Affiliations:** Department of Pharmacology and Toxicology, National Veterinary Research Institute (NVRI), al. Partyzantów 57, 24-100 Puławy, Poland; iwona.szymanek@piwet.pulawy.pl (I.S.-B.); aposyn@piwet.pulawy.pl (A.P.)

**Keywords:** polypeptide antibiotics, liquid chromatography, mass spectrometry, animal tissues, validation

## Abstract

A novel UHPLC-MS/MS method for the determination of polypeptide antibiotic residues in animal muscle, milk, and eggs was developed and validated. Bacitracin A, colistin A, colistin B, polymyxin B1, and polymyxin B2 were extracted from the samples with a mixture of acetonitrile/water/ammonia solution 25%, 80/10/10 (*v*/*v*/*v*), and put through further evaporation, reconstitution, and filtration steps. The chromatographic separation was performed on a C18 column in gradient elution mode. Mass spectral acquisitions were performed in selective multiple reaction monitoring mode by a triple quadrupole mass spectrometer. The method was validated according to the criteria of Commission Decision 2002/657/EC. The method quantifies polypeptides in a linear range from 10 to 1000 μg kg^−1^, where the lowest concentration on the calibration curve refers to the limit of quantification (LOQ). The recoveries ranged from 70 to 99%, the repeatability was below 13%, and within-laboratory reproducibility was lower than 15%. The decision limit (CCα) and detection capability (CCβ) values were calculated, and ruggedness and stability studies were performed, to fulfill the criteria for confirmatory methods. Moreover, the developed method may also be used for screening purposes by its labor efficiency.

## 1. Introduction

**Figure 1:**
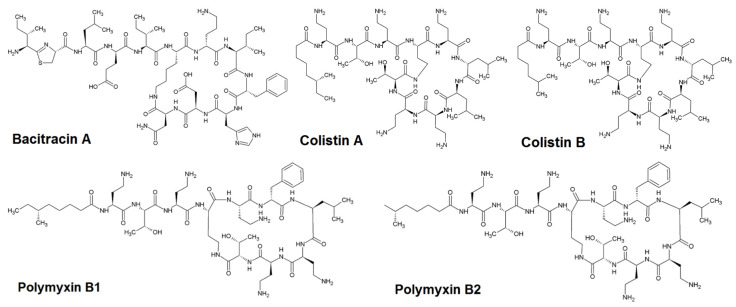
Chemical structures of polypeptide antibiotics.

Polypeptide antibiotics are a group of antimicrobials with a variety of actions against many Gram-negative and Gram-positive bacteria. Members of the polypeptide family are bacitracin, colistin A, colistin B, polymyxin B1, and polymyxin B2. These large-molecular-mass compounds have a common structure of a heptapeptide ring with a polypeptide side chain (Figure 1).

Bacitracin is produced by *Bacillus licheniformis* and *Bacillus subtilis* and is a mixture of several closely related polypeptides, mainly consisting of bacitracin A (above 50%), and of bacitracin B1, B2, C, and F to a lower extent [1]. Colistin (also known as polymyxin E) is an important member of the polymyxin group of cationic peptide antibiotics and is produced by cultures of *Bacillus polymyxa* var. *colistinus*. At least thirty different components have been found in commercially available colistin. The major components are colistin A (polymyxin E1) and colistin B (polymyxin E2), which differ by a single carbon in the fatty acyl moiety and account for more than 85% of the total colistins used in pharmaceutical products [2]. Polymyxin B is also derived from *Bacillus polymyxa*, and it is a mixture of over thirty polymyxin B polypeptides. Polymyxin B1 and B2, which differ by a single carbon in the fatty acyl moiety, are the two major components that account for >75% polypeptide mixture [3].

Due to their high toxicity, polypeptides are restricted in use in human medicine, but these antibiotics have been widely utilized as veterinary drugs and feed additives in animal husbandry [4]. In 2016, polymyxins (colistin only) were the fifth most-sold group of antimicrobials for food-producing animals in 30 European countries (29 EU/EEA) [5]. Despite the advantages of polypeptides in livestock production, their abuse in feed and the ignorance of their withdrawal time may result in drug residues in animal-derived food. Moreover, the long-term use of colistin in animal husbandry has led to the occurrence of a plasmid-mediated colistin resistance mechanism (mcr-1), meaning that multidrug-resistant bacterial infection might not be responsive to medical treatment, even with polymyxins as last-resort drugs [6]. Therefore, to ensure human food safety, the EU has set a tolerance level for these compounds as the maximum residue limit (MRL). The European Commission regulation 37/2010/EU [7] has set MRLs for colistin at the level of 50 µg kg^−1^ in milk, 150 µg kg^−1^ in muscle, and 300 µg kg^−1^ in eggs. In the case of bacitracin, MRLs have been established only for milk (100 µg kg^−1^) and muscle (150 µg kg^−1^). For polymyxin B, the MRLs have not been established for any sample type from any food-producing species.

Over the past years, several LC-MS/MS methods have been published for the analysis of polypeptide antibiotics in food of animal origin. Sin et al. [8] developed a quantitative method for the detection of bacitracin A and colistin A in bovine samples. Later, the same research group [9] extended the scope of the method by including the analysis of colistin B in poultry and porcine tissues. Xu et al. [10] published a method for colistin A and B in fishery products, and Zhang et al. [11] developed a method for the determination of bacitracin and polymyxin B in livestock products. Recently, two methods were developed for the determination of colistin A and B in animal tissues [12,13]. Meanwhile, there are only a few multi-residue methods for the determination of polypeptide antibiotics in animal matrices. Kaufman and Widmer [14] developed a multi-residue method for five polypeptide antibiotics in a variety of food matrices. In turn, Boison and coworkers [15] developed a method for the determination of seven polypeptides; however, this method only applies to chicken muscles.

The aim of this work was to develop a reliable UHPLC-MS/MS method with fast and simple sample pretreatment, suitable for extraction of polypeptide residues from muscle, milk, and egg samples. The sample preparation and instrument conditions were optimized. One of the novelties of this method is using a mixture of acetonitrile/water/ammonia solution 25%, 8/1/1 (*v*/*v*/*v*), as an extraction solvent, in contrast to the commonly used acidic extraction followed by solid phase extraction. To conclude the development process, the method was validated in accordance with the rules in Commission Decision 2002/657/EC [16] and demonstrated to be suitable for the detection and quantitation of polypeptide antibiotic residues in food of animal origin.

## 2. Results and Discussion

### 2.1. Optimization of LC-MS/MS Conditions

The analysis of peptides was mostly carried out in reversed-phase LC-MS/MS detection, using an electrospray (ESI) interface [17]. ESI in positive mode was the ionization technique of choice for the LC-MS/MS analysis of polypeptides because protonation is favored by the presence of amine groups. However, LC-MS/MS assays tend to have a lower sensitivity for peptides than for small molecules because of multiple peptide charge states, isotopic distribution, and a higher degree of fragmentation [18].

**Figure 2:**
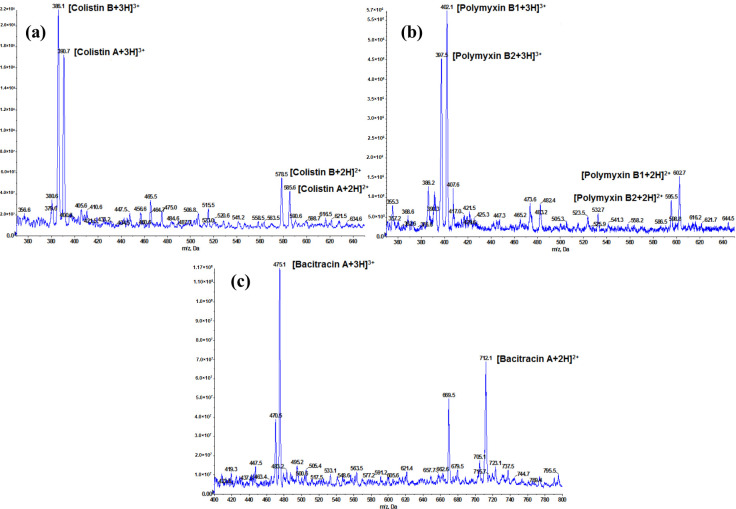
Mass spectrum of polypeptide antibiotic in which doubly and triply charged ions of colistin A and B (**a**); polymyxin B1 and B2 (**b**); and bacitracin A (**c**) can be seen.

In this work, the mass parameters were optimized by infusing standard solutions of polypeptide antibiotics (100 ng mL^−1^). A full scan in ESI positive-ionization mode was performed to select the most abundant precursor ion. For all analytes, doubly charged [M + 2H]^2+^ and triply charged ions [M + 3H]^3+^ were observed, and the intensity of singly charged ions [M + H]^+^ was very weak. Because [M + 3H]^3+^ ions (bacitracin A at *m*/*z* 475, colistin A at *m*/*z* 390.7, and B at *m*/*z* 386, polymyxin B1 at *m*/*z* 402, and B2 at *m*/*z* 397.5) were the most intense, they were selected as precursor ions to provide the best assay sensitivity (Figure 2).

The fragmentation reactions used for the monitoring of polypeptide antibiotics were selected on the basis of their significance in product ion spectra. Two multiple-reaction monitoring (MRM) transitions of polypeptides were examined in order to isolate and optimize ion selection and fragmentation and increase sensitivity, fulfilling the criteria of the European Union regarding unequivocal identification [16]. The first transition was chosen for the quantification, and the second was used for the confirmation. Analyte identification was carried out by retention time and relative ion ratio of selected MRM transition. The optimal conditions for the detection of polypeptides by LC-MS/MS are reported in Supplementary Materials Table S1. MS/MS-determined fragments, as obtained in the present work, are consistent with those reported in the literature [8,9,12,19,20,21].

Chromatography of peptide drugs is also a challenge. The most common issues encountered during peptide chromatography are peak shape, separation, and carryover. In order to obtain sharp and symmetrical peaks on a C18 column, acidified mobile phases with different concentrations of formic acid [9,10,11,12,15] and/or trifluoroacetic acid were used [14]. The advantage of low pH is the suppression of ionization of silanol groups, thereby limiting secondary interaction with cationic compounds and thus preventing peak tailing. Although trifluoroacetic acid can significantly improve peak shape, it also suppresses MS ionization and reduces overall sensitivity.

**Figure 3:**
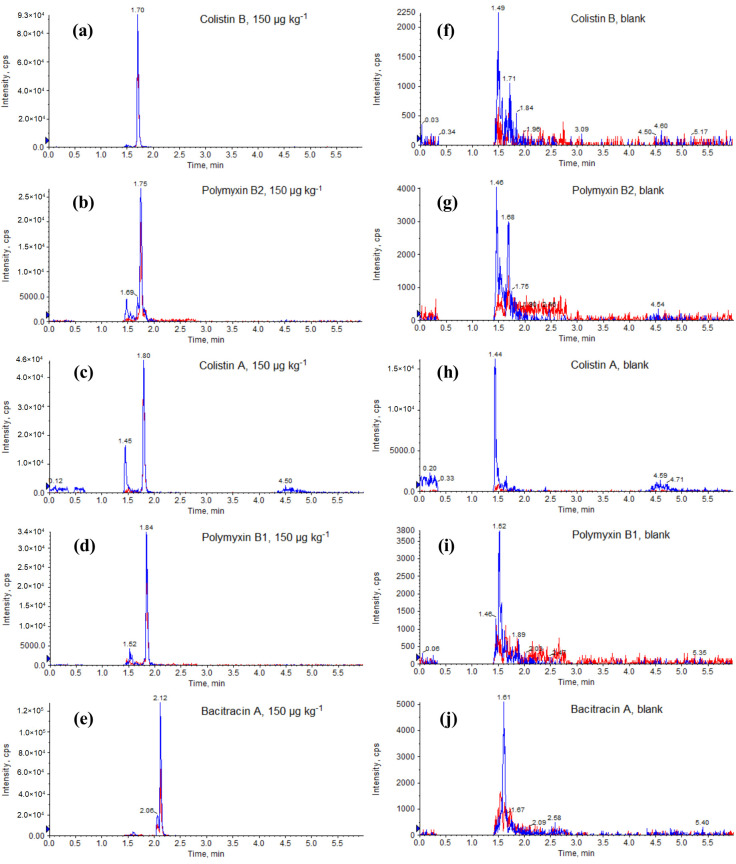
UHPLC-MS/MS chromatograms in multiple-reaction monitoring (MRM) mode of a fortified swine muscle sample at 150 µg kg^−1^ (**a**–**e**) and a blank swine muscle sample (**f**–**j**).

During optimization, we tested different C18 chromatographic columns and mobile phases for the separation of polypeptide antibiotics. To investigate the effect of the acid modifier, we tested the mobile phase composition with different concentrations of formic acid (0.1–1%). The optimal UHPLC separation of analytes was obtained on a Kinetex 2.6 μm XB-C18 column with a mobile phase consisting of 1% formic acid in acetonitrile and 1% formic acid in water and the gradient program described later (Section 3.2). Using these conditions, we obtained sharp symmetrical chromatographic peaks with minimum band broadening. Ion chromatograms obtained from muscle samples spiked with polypeptides at 150 µg kg^−1^ are presented in Figure 3. Even though, under these conditions, full separation between some analytes was not achieved, the ultimate LC separation is not required, because sufficient specificity is provided by the MS/MS (unique MRM transitions of each polypeptide). Potential column contamination was prevented through the use of the mobile phase gradient; although the retention time of the polypeptide was <2.20 min, the gradient continued for 6 min, to ensure that all elutable components were cleared and the system was re-equilibrated prior to injecting the next sample. Unlike other LC-MS/MS methods [8,9,11,14,15], the time of chromatographic analysis is significantly shorter and comparable to those in which only colistins were determined [10,12]. Blank samples were also run to ensure that no carryover or matrix effects were present (Figure 3). In summary, we felt comfortable enough with the method and instrument ruggedness gained by UHPLC chromatography and this choice of MS/MS conditions.

### 2.2. Optimization of Sample Preparation

This sample preparation method was developed after reviewing the main published LC-MS/MS methods for the determination of polypeptide antibiotics in the food of animal origin. The sample preparation procedure principally involved a deproteinization step (referred to as acidic pretreatment), which is performed by organic solvents (methanol and acetonitrile) and acids (trifluoroacetic acid, trichloroacetic acid, and hydrochloric acid) in different combinations [8,9,10,11,12,13,14,15]. After that step, solid-phase extraction is implemented on polymeric-based SPE [9,10,11,14], C18-SPE [15], or ion exchange cartridges [12,13]. Finally, before LC-MS/MS analysis one of several different filtration steps occurs [8,10,11,12,15].

Our aim was to develop a new, simple, fast, and robust extraction and cleanup method which enables its users to determine polypeptide antibiotic residues in food of animal origin at quite low concentration levels. In contrast to the abovementioned LC-MS/MS methods, we decided to omit SPE step because it involves tedious procedures of sample loading, washing and elution, resulting in long handling time and loss of analytes. Furthermore, the high cost of commercial SPE cartridges is not appropriate for extensive analysis of polypeptide antibiotics in food samples. However, the most important novelty in sample preparation was the replacement of common acid extraction with a new extraction approach in an alkaline environment.

Polypeptide antibiotics are amphiprotic drugs, which have in their structure both weakly acidic (carboxylic acid) and weakly basic (amino) functional groups. Because peptides have both positive and negative charges (dipolar compounds) in aqueous solutions, they can exist in three forms: a protonated acid form, neutral form (at the isoelectric point), or deprotonated base form. On the basis of the physicochemical properties of peptide antibiotics, we wanted to change the ionization state of these analytes to a more apolar state in which extraction with organic solvent will be possible. For this reason, we chose acetonitrile (ACN), a polar aprotic solvent, because it is the most frequently reported extraction solvent for the analysis of drug residues in products of animal origin and typically provides high extraction recoveries, minimizes co-extraction of lipids, and is efficient for denaturation of proteins [22]. However, it is reported that ACN does not sufficiently extract polar analytes [23]. Additionally, we used 25% ammonia solution because it has alkaline properties (a weak base), can be mixed with ACN, and is volatile, so it evaporates reasonably quickly.

**Figure 4:**
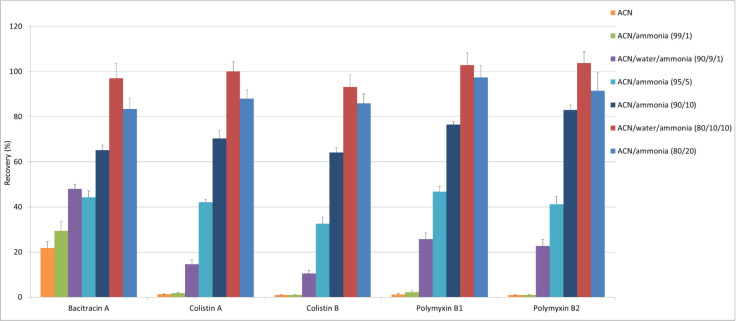
The extraction efficiency of polypeptide antibiotics in different compositions of extraction solvent.

Optimization of sample preparation concerned both the selection of appropriate proportions of extraction mixture components and the individual stages of sample handling (shaking, sonication, evaporation, and filtration) to obtain the sample treatment that was as short as possible, while having acceptable recoveries. To optimize the extraction procedure, seven extraction solvents consisting of different ratios of 25% ammonia solution and ACN were compared (Figure 4). All experiments were performed in quadruplet by spiking blank muscle samples with analytes at 150 µg kg^−1^. We chose muscle tissue for the optimization of sample treatment procedures because it is the most frequently consumed tissue with the highest content of proteins. Extraction efficiency (expressed as recovery) for all analytes were estimated without correction for the losses during the sample preparation, by comparing the peak area of each compound in the samples spiked at the beginning of extraction against the peak area in the matrix-matched standards (final extract of blank samples by which the antibiotics were added immediately before LC injection). In this way, we calculated the yield of the extraction stage (the recovered quantity of analyte from the spiked sample) compensated by matrix effect.

Given the polarity of polymyxins, extraction with pure ACN was not possible, and only bacitracin A was recovered at about 20%. After the addition of ammonia, we observed the gradually increasing effectiveness of the extraction for all of the analytes, especially in the case of polymyxins. Finally, the best effects on the recovery for all polypeptides were achieved after sample treatment with the mixture of ACN/water/ammonia solution 25%, 80/10/10 (*v*/*v*/*v*). This was caused not only by the incremental rise in ammonia content, but also the increasing water content in the extraction mixture. This is clearly seen when we compare results with those gained with a similar ammonia content, but different water content (i.e., ACN/ammonia solution 25%, 99/1 (*v*/*v*), vs. ACN/water/ammonia solution 25%, 90/9/1 (*v*/*v*/*v*), or ACN/ammonia solution 25%, 90/10 (*v*/*v*), vs. ACN/water/ammonia solution 25%, 80/10/10 (*v*/*v*/*v*). However, an explanation of the mechanism of this extraction is not easy, because the extraction solvent is a mixture of polar reagents, some aprotic (ACN) and others protic (water or ammonia), and the physicochemical properties of peptides (Supplementary Materials Table S2) are derived from water solutions. In the literature, there are no studies about the solubility of polypeptide antibiotics in water/organic solvent mixtures. There are only reports of the solubility of amino acids in water at various pH levels [24,25], in ACN/water [26], or ethanol/water systems [27]. Therefore, based on empirical observation, we can hypothesize that, in alkaline medium, our analytes having isoelectric points at pH 8.06 (bacitracin A), 10.41 (colistin A and B), and 10.42 (polymyxin B1 and B2) [28] are in deprotonated base form, and for that reason, effective extraction (about 90%) of them from the matrix could be possible.

After optimization of extraction mixture, we tested rotatory stirrer and ultrasonic bath usage in different time intervals (10, 20, and 30 min) on extraction efficiency. Since we did not observe any significant differences in the results obtained along with the increase of extraction time, we assumed that the optimal time of these stages is 10 min. On the other hand, we can conclude that our analytes are stable in an alkaline environment (extraction mixture) for at least the duration of the extraction process. In the next step, we tested different volumes of extraction solvent (6, 8, and 10 mL). Obtained results showed that recoveries after extraction with 6 mL of extraction mixture were slightly lower (about 10%) than those obtained after extraction by 8 and 10 mL. However, after extraction with 10 mL, the time of evaporation notably increased, so we decided to use 8 mL of extraction solvent in our final method. Moreover, to speed up the evaporation step, we tested the different temperature of evaporation (40, 45, and 50 °C). The results of this study showed that the best option is evaporation at 45 °C. Although at 40 °C the recoveries were comparable to those obtained at 45 °C, under these conditions, it significantly increases the overall analysis time. In turn, at 50 °C, the extract evaporated faster, but on the other hand, lower recoveries were observed probably due to partial degradation of the analytes. Lastly, to reduce the possible interfering components in the final extract, three different syringe filter membranes (PVDF, PTFE, and nylon) were tested. The usage of PVDF filters allowed us to achieve the best results for all analytes, without recoveries’ reduction. Overall, this combination of steps of sample preparation provided clean extract and optimum levels of analyte recovery. Finally, the optimized sample preparation was tested on different matrices (milk and eggs), where the recoveries were also acceptable (above 70%). Thus, these matrices were also included in the developed procedure.

### 2.3. Method Validation

The whole procedure was validated, as stipulated in Commission Decision 2002/657/EC [16]. Because isotope-labeled analytical polypeptide standards were not available, we employed external standard quantitation. Moreover, standard substances of bacitracin that would allow us to determine the marker residue (sum of bacitracin A, bacitracin B, and bacitracin C) were not available [7]. We were able to purchase only the standard of bacitracin A, which is the main component of bacitracin (more than 50%) [1]. However, taking into account the fact that other authors of LC-MS/MS methods for the determination of bacitracin in food samples quantify only bacitracin A [8,9,11,14,15], we decided to apply their interpretation of marker residue, as well as the interpretation of MRL for muscle [14]. Moreover, we were able to obtain only a colistin reference substance (colistin sulfate salt), which consisted of a mixture of colistin A and B. The same situation occurred with the reference standard of polymyxin B (polymyxin B sulfate), which consists of a mixture of polymyxin B1 and B2. To complicate this issue, the proportions of colistin A and B and polymyxin B1 and B2 in reference materials differ between batches and manufacturers. For this reason, we calculated the percentages of colistin A and B in a reference sample of colistin as the ratios of peak areas for both and found them to be 39 and 61, respectively. The same was carried out for the polymyxin reference material, where we calculated the percentages of polymyxin B1 and polymyxin B2 at 63 and 37, respectively. The calculations were based on the assumption that the instrumental response factor for colistin A and B, as well as for polymyxin B1 and B2, are the same because they differ only in single carbon in the fatty acyl moiety. A similar approach is reported in the literature [9,29]. Therefore, we decided that the quantification calculation given in the validation data is based on the assumption that the reference substance of colistin and polymyxin B contains 100% of each of colistin A, colistin B, polymyxin B1, and polymyxin B2.

The specificity of the method was checked by analyzing 20 blank samples of each matrix. No endogenous interference was observed at the mass transitions of each target compound within the 2.5% margin of the retention time, indicating the good specificity of the developed method. The linearity in the solvent and the matrix was evaluated to lie in the range 10–1000 μg kg^−1^, with coefficients of determination (r^2^) higher than 0.99 for all analytes. Recovery (trueness), repeatability and within-laboratory reproducibility (precision), decision limit (CCα), detection capability (CCβ), and matrix effect were calculated for muscle (Table 1), milk (Table 2), and eggs (Table 3). The recoveries in muscle ranged from 91 to 99%, in milk from 78 to 98%, and in eggs from 70 to 83%. These results obtained for muscle, milk (except for polymyxin B1) and eggs (only bacitracin A) fulfills requirements of minimum trueness (80–110%) [16]. Although the recoveries obtained for polymyxin B1 in milk and for colistins and polymyxins in eggs were slightly below 80%, these results are comparable to those obtained by Kaufmann and Widmer [14], who also used external calibration for calculations. To obtain high recoveries, other authors for quantification used internal standards like polymyxin B [8,9] and colistin [11] or matrix fortified calibration standards [15], which inherently correct recoveries. However, in this kind of quantitation of “apparent recovery”, when we obtain a 100% recovery, it does not mean that we have a 100% yield for any extraction or preconcentration step. Results from the assessment of repeatability and from the within-laboratory reproducibility study (CV 6.0–12.7% and 7.7–13.9%, respectively) show that the precision of the determination of polypeptide antibiotics was acceptable (CV below 16%). Taking into account the results from recovery and precision study, we can conclude that method accuracy is satisfactory. The calculated CCα and CCβ values were comparable to those reported by other researchers [13,14]. Although the CD 2002/657/EC does not require the estimation of LOQ, we determined LOQ to characterize the sensitivity and compared it with other methods. On the other hand, the Document SANCO/2004/2726-rev 4 recommends to determine, also for substances for which MRLs have been set, the lowest detectable concentration level and the accuracy at that concentration (LOQ in our case) [30]. Therefore, for authorized compounds, we also established LOQ, because between CCα and LOQ, we may find samples judged to be suitable for human consumptions (MRL is not exceeded), but deriving from an illegal or unregistered animal treatment farm. The sensitivity of the method was satisfactory, which can be confirmed by the low LOQ (10 μg kg^−1^) for all analytes in all matrices. The LOQ values of the developed method were lower than those of polymeric-based SPE (20–200 μg kg^−1^ [9], 40 μg kg^−1^ [10], 30–250 μg kg^−1^ [11]), or C18-SPE (30–74 μg kg^−1^) [15] sample preparation methods. Only one method reported lower LOQ for colistin A and B (5–30 μg kg^−1^) [12] and other similar values (10–33 μg kg^−1^) [13], as compared to our LOQ for these analytes. This may be due to the application of a more selective purification of the extract with ion exchange SPE cartridges, unlike with the early mentioned SPE methods. The matrix effect that is produced by matrix components co-extracted with analytes causes signal suppression or enhancement during the ESI ionization. Therefore, it is important to eliminate or compensate for this effect, in order to achieve reliable results. It was observed that the matrix effect on the response of the analyte depended on the type of tissue. More considerable matrix effects (ion suppression) were measured in muscle (from −66 to −48%) than in milk (from −65 to −33%) or egg (from −34 to −25%). Similarly, other researchers have also failed to eliminate the matrix effect, even with more sophisticated purification of the extract by SPE [10,12,13,14,15]. Even though the matrix effect was not eliminated, the use of matrix-matched calibration standard curves allowed satisfactory accuracy to be reached. Method ruggedness (minor changes) was estimated by using the Youden approach [16]. The experimental design was planned to identify seven different variables in the sample preparation procedure (Supplementary Materials Table S3). The effect of each of the factors during the preparation of the muscle sample was evaluated (Supplementary Materials Table S4). As the obtained value of the standard deviation of difference (SD_i_) was less than the standard deviation of the within-laboratory reproducibility (SD_WLR_), it was demonstrated that all selected factors together do not significantly affect the analytical performance. Besides this general result, the influence of each variable on method ruggedness was also evaluated by applying the *t*-test. Experimental *t*-values were always lower than *t*_crit_, demonstrating the method’s ruggedness for slight variations of selected parameters. Consequently, the method proved to be fairly robust and able to withstand minor fluctuations in the operating variables that may occur during the sample preparation. The test for stability of analytes in solutions showed that the standard solutions stored < −18 °C were stable for at least one month. The stability of mixed standard solutions stored in a refrigerator (2–10 °C) was maintained for at least one week.

## 3. Materials and Methods

### 3.1. Chemical and Reagents

All of the solvents used were of analytical grade. ACN and methanol were obtained from J.T. Baker (Deventer, the Netherlands). Ammonia solution 25% was purchased from POCH (Gliwice, Poland). Formic acid was obtained from Honeywell Fluka (Seelze, Germany). Ultra-pure water (resistance >18 mΩ) was obtained from a Milli-Q system (Millipore, Molsheim, France). Syringe filters that were 0.22 μm in size and PVDF material were from Restek (Bellefonte, PA, USA). The analytical reference standards of bacitracin A, colistin sulfate salt, and polymyxin B sulfate were bought from Sigma-Aldrich (St. Louis, MO, USA).

Stock standard solutions (1000 μg mL^−1^) of analytes were prepared separately by weighing 10 mg of reference standard and dissolving this in 10 mL of methanol (bacitracin A) or 10 mL of a mixture of methanol/0.1% formic acid in water, 1/1 (*v*/*v*), in the cases of colistin and polymyxin B. The stock solutions were stored in dark glass bottles, at < −18 °C, for 1 month. The working standard solutions used for sample fortification were prepared by the dilution of these solutions with water and were stored in the dark, at 2–10 °C, for one week. The outdated standard solutions were disposed of in compliance with all pertinent legislation.

### 3.2. LC-MS/MS Conditions

A Shimadzu Nexera X2 UHPLC system (Shimadzu, Kyoto, Japan) was connected to a QTRAP 4500 mass spectrometer (AB Sciex, Concord, ON, Canada). Analyst 1.6 software (AB Sciex, Concord, ON, Canada) controlled the LC-MS/MS system and processed the data. The mass spectrometer was operated in the electrospray positive ionization mode (ESI+), and the multiple-reaction monitoring (MRM) mode was used to quantify the analytes. The mass spectrometer settings were optimized, and the following parameters were used: Q1 and Q3 resolution—unit; curtain gas—20; collision gas—medium; ion source gas 1—50; ion source gas 2—60; ion spray voltage—4500 V; and ion source temperature—400 °C.

The chromatographic separation was performed on a Kinetex 2.6 μm XB-C18 column (2.1 × 100 mm, Phenomenex, Torrance, CA, USA) coupled with a SecurityGuard ULTRA holder and cartridge UHPLC C18 for 2.1 mm ID columns (Phenomenex, Torrance, CA, USA). The mobile phase consisted of solvent A, 1% formic acid in ACN, and solvent B, 1% formic acid in water. The elution was performed in a gradient mode. The starting condition for the mobile phase was 95% of eluent B, and then eluent B was decreased to 5% within 3.50 min. Next, this condition was maintained for 1 min, to completely elute matrix components. At 4.51 min, eluent B was returned to the initial 95%, and with the subsequent equilibration time of 1.50 min, the resulting total run was 6 min. The column was operated at 45 °C with a flow rate of 0.40 mL min^−1^, and the injection volume was 10 μL.

### 3.3. Sample Preparation

Animal muscles were obtained from slaughterhouses (Lublin Province, Poland). Milk and egg samples were purchased from local supermarkets (Puławy, Poland). Muscle and egg were homogenized in domestic blenders, and all samples were stored in freezers, at < −18 °C, prior to analysis. A 2.00 ± 0.01 g of the homogenized sample was weighed into a 50 mL polypropylene centrifuge tube. Then, 8 mL of ACN/water/ammonia solution 25%, 80/10/10 (*v*/*v*/*v*), was added to the tube; the sample was vigorously mixed on a vortex mixer for about 1 min, shaken for about 10 min on the rotary stirrer, and sonicated for 10 min. After the centrifugation at 4500 rpm for 10 min at 4 °C, the supernatant was taken and placed in a 15 mL tube. The extract was evaporated to dryness, under a weak stream of nitrogen, at 45 °C. The dry residue was reconstituted in 1 mL of a mixture of 1% formic acid in water/1% formic acid in ACN, 95/5 *(v*/*v)*, and sonicated for 5 min. Next, the extract was centrifuged at 4500 rpm for 10 min at 4 °C and filtered through a 0.22 μm PVDF syringe filter into an LC vial for UHPLC-MS/MS analysis.

### 3.4. Method Validation

The method was validated according to the rules of the Commission Decision 2002/657/EC [16] that establish the validation guidelines and general and numeric criteria for evaluation of fitness for purpose of a method for residue analysis. To evaluate possible interferences encountered in the method, the specificity was validated by analyzing different blank samples of muscle (poultry, swine, bovine, and rabbit), milk (bovine), and egg (chicken) from different origins. The linearity in the solvent (1% formic acid in water/1% formic acid in ACN, 95/5 (*v*/*v*)) and in the matrix was evaluated in the range of 10–1000 μg kg^−1^. The matrix-matched solutions were prepared by extracting blank (i.e., drug-free) tissue samples, following the method described, and adding the appropriate analyte standards to the extract, just prior to reconstitution with solvent immediately before the LC injection (Supplementary Materials Table S5). The curve was constructed by plotting the average peak area of the analyte (taken from three injections for each concentration point) against its concentration. Method trueness and precision were evaluated according to the matrix-matched approach, by analyzing blank samples spiked at the beginning of the extraction procedure at 10 μg kg^−1^, 0.5, 1.0, and 1.5 times the MRL level, with the appropriate standard solutions (Supplementary Materials Table S5). Since MRLs have not been set for bacitracin in egg and for polymyxin B in all matrices, the fortification levels for these compounds duplicated the concentration levels for the analytes with MRLs values (cascade option). In the repeatability study, four series were analyzed under identical conditions (six samples for each fortification level). Each level was made the subject of standard deviation (SD) and coefficient of variation (CV, %) calculations. The within-laboratory reproducibility was obtained by analysis of two additional series (at the four fortification levels), under reproducibility conditions (two different occasions and different technicians), and the overall SD and CV were calculated. Taking the overall mean concentrations obtained in the reproducibility study, trueness was calculated and expressed in terms of recovery as a percentage and precision as relative standard deviation (CV, %). The CCα and CCβ parameters were calculated according to the calibration curve procedure reported in Commission Decision 2002/657/EC [16] and clarified in the document SANCO/2004/2726-rev 4 [30]. In the case of polypeptides with the MRLs, CCα was calculated as the mean measured concentration at the MRL of each compound plus 1.64 times the SD of within-lab reproducibility at this concentration. CCβ was calculated as CCα plus 1.64 times the SD of within-lab reproducibility at CCα. For substances for which there is no permitted limit (without MRLs), document SANCO/2004/2726-rev 4 states that CCα and CCβ should be as low as reasonably achievable. However, it is widely recognized that, where the matrix calibration curve procedure described in the Commission Decision 2002/657/EC was used to calculate CCα for which no permitted limit has been established, the values obtained may be too low to be confirmed experimentally. In order to avoid these extrapolated theoretical values, we calculated CCα by using parallel extrapolation to the *x*-axis at the lowest experimental concentration [30]. CCα was expressed as the concentration corresponding to the lowest calibration level (10 μg kg^−1^) plus 2.33-fold the SD of within-lab reproducibility at this level. CCβ was calculated as CCα + 1.64-fold the SD of the within-lab reproducibility at CCα. Therefore, CCα and, consequently, CCβ achieved with this approach were determined inside the experimental validation range and not extrapolated. The LOQ (defined as the lowest concentration of the analyte that could be determined with accuracy) was estimated on the basis of the observed recovery and precision at the first validation concentration (10 μg kg^−1^). Matrix effects (ME) were calculated by comparison of the slopes of matrix-matched curves with the same curves prepared in solvent (without matrix), expressed as Equation (1):

(1) $$ME\left(\%\right)=\frac{Slope\;of\;matrix-matched\;standard\;curve-Slope\;of\;standard\;curve}{Slope\;of\;standard\;curve}\times 100\%$$

The positive values of ME indicate signal enhancements, while the negative indicates signal suppression [31]. Ruggedness was evaluated according to the Youden approach, by adopting the experimental design described in Decision2002/657/EC. Eight experiments were carried out in order to estimate the effect on method ruggedness of minor changes in seven variables selected from the sample preparation procedure. The details are listed in Supplementary Materials Table S3. The experiments were carried out by determination of spiked blank muscle, with all polypeptides at the intermediate validation level (150 μg kg^−1^), and their recovery was checked. The results were statistically evaluated by *t*-test and comparison of standard deviation of differences (SD_i_) and standard deviation of within-laboratory reproducibility (SD_WLR_). Stock standard solutions (1000 μg mL^−1^) and working standard solution (2 μg mL^−1^) were used for the verification stability of the analytes. The stock solutions were stored at < −18 °C, and working standard solution was stored in the refrigerator (2–10 °C), for up to 6 months and 4 weeks, respectively. After 1, 3, and 6 months (stock solutions), or 1, 2, and 4 weeks (working standard solution), the average peak areas of tested solutions (after dilution to 300 ng mL^−1^) were compared to the average peak area of standard solution kept in reference condition (< −70 °C) from the beginning of experiment (*t* = 0). If the average peak area at a certain storage time was above 90% of the average peak area at *t* = 0, the compound was considered stable for that specific storage time. If it dropped below 90%, the solution was considered to be unstable.

## 4. Conclusions

An analytical method for the determination of polypeptide antibiotics in muscle, milk, and egg samples was successfully developed. A simple sample preparation and 6 min chromatographic run allowed multiple analyses to be performed within one working day. To the best of our knowledge, this is the first simple method based on alkaline extraction of polypeptide antibiotics in food of animal origin. This is an important novelty over studies that employ acidic extraction followed by solid phase extraction. The method validation parameters demonstrate its reliability, high recovery and precision, and good specificity. Moreover, the developed method fulfills the criteria for confirmatory methods and may be used also for screening purposes by its labor efficiency. In summary, this method is practical, economical, and efficient for the simultaneous determinations of trace amounts of polypeptides antibiotics in food of animal origin.

## Figures and Tables

**Table 1:** Validation results of the method for the determination of polypeptides in muscle.

Parameter	Bacitracin A	Colistin A	Colistin B	Polymyxin B1	Polymyxin B2
Spiked level (µg kg^−1^)	10/75/150/225	10/75/150/225	10/75/150/225	10/75/150/225	10/75/150/225
Recovery (%)	91/93/95/95	97/95/94/96	94/93/94/97	99/93/95/95	97/93/94/96
Repeatability (CV, %)	9.8/7.2/6.8/6.3	9.7/7.9/7.8/7.4	9.0/7.3/6.9/6.0	9.9/9.7/8.8/8.5	9.8/9.3/8.6/7.7
Within-lab reproducibility (CV, %)	10.4/9.0/8.6/7.7	12.6/10.4/10.5/9.4	10.4/10.1/9.5/8.4	12.0/11.0/9.9/9.7	12.2/10.2/9.6/8.8
CCα (µg kg^−1^)	168	172	177	14.3	13.6
CCβ (µg kg^−1^)	192	203	206	17.8	16.7
Matrix effect (%)	−62	−49	−48	−66	−65

**Table 2:** Validation results of the method for the determination of polypeptides in milk.

Parameter	Bacitracin A	Colistin A	Colistin B	Polymyxin B1	Polymyxin B2
Spiked level (µg kg^−1^)	10/50/100/150	10/25/50/75	10/25/50/75	10/25/50/75	10/25/50/75
Recovery (%)	96/98/98/96	89/84/83/85	86/82/79/78	79/78/78/81	80/81/80/83
Repeatability (CV, %)	11.1/9.3/9.0/8.9	11.6/10.2/9.8/8.9	11.3/10.6/10.5/9.7	12.4/12.2/10.4/9.7	12.7/10.8/10.0/9.5
Within-lab reproducibility (CV, %)	13.4/10.0/10.2/9.6	13.7/12.3/12.0/10.5	13.3/11.2/11.0/10.4	13.9/13.1/11.6/10.5	13.4/12.0/10.6/10.3
CCα (µg kg^−1^)	117	60.5	59.0	13.1	13.1
CCβ (µg kg^−1^)	140	73.7	71.8	16.6	16.5
Matrix effect (%)	−65	−35	−33	−38	−41

**Table 3:** Validation results of the method for the determination of polypeptides in eggs.

Parameter	Bacitracin A	Colistin A	Colistin B	Polymyxin B1	Polymyxin B2
Spiked level (µg kg^−1^)	10/150/300/450	10/150/300/450	10/150/300/450	10/150/300/450	10/150/300/450
Recovery (%)	83/80/83/83	71/74/72/73	71/75/75/74	70/75/74/73	72/74/72/72
Repeatability (CV, %)	10.4/9.7/8.2/7.0	10.8/7.7/7.3/7.2	11.5/7.1/6.9/6.5	10.5/8.1/7.9/7.3	9.2/7.2/6.9/6.8
Within-lab reproducibility (CV, %)	11.2/11.5/9.6/8.8	12.1/10.5/9.8/9.6	13.2/10.7/9.6/8.8	12.0/11.5/10.3/9.7	11.2/10.1/8.7/8.7
CCα (µg kg^−1^)	13.9	340	343	14.3	14.1
CCβ (µg kg^−1^)	18.4	402	400	18.3	17.9
Matrix effect (%)	−25	−28	−26	−29	−34

## Supplementary Materials

The following are available online. Table S1: LC-MS/MS parameters used for monitored compounds. Table S2: Some physicochemical properties of studied compounds [28]. Table S3: Variables and their levels in the Youden ruggedness test experimental design. Table S4: Statistical evaluation of ruggedness test results (seven factors, eight experiments) for muscle samples. Table S5: Preparation of the calibration curves and the spiked samples.

## References

[1] The European Agency for the Evaluation of Medical Products Committee for Medicinal Products for Veterinary Use. Bacitracin. Summary Report (2). EMEA/MRL/768/00-FINAL, January 2001. https://www.ema.europa.eu/en/documents/mrl-report/bacitracin-summary-report-2-committee-veterinary-medicinal-products_en.pdf.

[2] Ma Z., Wang J., Gerber J.P., Milne R.W. (2008). Determination of colistin in human plasma, urine and other biological samples using LC-MS/MS. J. Chromatogr. B Analyt. Technol. Biomed. Life Sci..

[3] Covelli J., Ruszaj D., Straubinger R., Li J., Rao G.G. (2017). The development and validation of a simple liquid chromatography-tandem mass spectrometry method for polymyxin B1 and B2 quantification in different matrices. J. Chromatogr. B Analyt. Technol. Biomed. Life Sci..

[4] Catry B., Cavaleri M., Baptiste K., Grave K., Grein K., Holm A., Jukes H., Liebana E., Lopez Navas A., Mackay D. (2015). Use of colistin-containing products within the European Union and European Economic Area (EU/EEA): Development of resistance in animals and possible impact on human and animal health. Int. J. Antimicrob. Agents.

[5] European Medicines Agency, European Surveillance of Veterinary Antimicrobial Consumption, 2018. Sales of Veterinary Antimicrobial Agents in 30 European Countries in 2016. https://www.ema.europa.eu/en/documents/report/sales-veterinary-antimicrobial-agents-30-european-countries-2016-trends-2010-2016-eighth-esvac_en.pdf.

[6] Liu Y.Y., Wang Y., Walsh T.R., Yi L.X., Zhang R., Spencer J., Doi Y., Tian G., Dong B., Huang X. (2016). Emergence of plasmid-mediated colistin resistance mechanism MCR-1 in animals and human beings in China: A microbiological and molecular biological study. Lancet Infect. Dis..

[7] European Communities (2010). Commission Regulation (EU) No 37/2010 of 22 December 2009 on pharmacologically active substances and their classification regarding maximum residue limits in foodstuffs of animal origin. Off. J. Eur. Communities.

[8] Sin D.W.M., Ho C., Wong Y.C., Ho S.K., Ip A.C.B. (2005). Analysis of major components of residual bacitracin and colistin in food samples by liquid chromatography tandem mass spectrometry. Anal. Chim. Acta.

[9] Wan E.C.H., Ho C., Sin D.W.M., Wong Y.C. (2006). Detection of residual bacitracin A, colistin A, and colistin B in milk and animal tissues by liquid chromatography tandem mass spectrometry. Anal. Bioanal. Chem..

[10] Xu Y., Tian X., Ren C., Huang H., Zhang X., Gong X., Liu H., Yu Z., Zhang L. (2012). Analysis of colistin A and B in fishery products by ultra performance liquid chromatography with positive electrospray ionization tandem mass spectrometry. J. Chromatogr. B.

[11] Zhang D., Park J.A., Kim D.S., Kim N.H., Kim S.K., Cho K.S., Jeong D., Shim J.H., El-Aty A.M.A., Shin H.C. (2015). Simultaneous detection of bacitracin and polymyxin B in livestock products using liquid chromatography with tandem mass spectrometry. J. Sep. Sci..

[12] Fu Q., Li X., Zheng K., Ke Y., Wang Y., Wang L., Yu F., Xia X. (2018). Determination of colistin in animal tissues, egg, milk, and feed by ultra-high performance liquid chromatography-tandem mass spectrometry. Food Chem..

[13] Saluti G., Diamanti I., Giusepponi D., Pucciarini L., Rossi R., Moretti S., Sardella R., Galarini R. (2018). Simultaneous determination of aminoglycosides and colistins in food. Food Chem..

[14] Kaufmann A., Widmer M. (2013). Quantitative analysis of polypeptide antibiotic residues in a variety of food matrices by liquid chromatography coupled to tandem mass spectrometry. Anal. Chim. Acta.

[15] Boison J.O., Lee S., Matus J. (2015). A multi-residue method for the determination of seven polypeptide drug residues in chicken muscle tissues by LC-MS/MS. Anal. Bioanal. Chem..

[16] European Communities (2002). Commission Decision (2002/657/EC) of 12 August 2002 implementing Council Directive 96/23/EC concerning the performance of analytical methods and the interpretation of results. Off. J. Eur. Communities.

[17] Rauh M. (2012). LC-MS/MS for protein and peptide quantification in clinical chemistry. J. Chromatogr. B Analyt. Technol. Biomed. Life Sci..

[18] Campbell J.L., Le Blanc J.C. (2011). Peptide and protein drug analysis by MS: Challenges and opportunities for the discovery environment. Bioanalysis.

[19] Suleiman S.A., Song F., Su M., Hang T., Song M. (2017). Analysis of bacitracin and its related substances by liquid chromatography tandem mass spectrometry. J. Pharm. Anal..

[20] Govaerts C., Rozenski J., Orwa J., Roets E., Van Schepdael A., Hoogmartens J. (2002). Mass spectrometric fragmentation of cyclic peptides belonging to the polymyxin and colistin antibiotics studied by ion trap and quadrupole/orthogonal-acceleration time-of-flight technology. Rapid Commun. Mass Spectrom..

[21] Govaerts C., Li C., Orwa J., Van Schepdael A., Adams E., Roets E., Hoogmartens J. (2003). Sequencing of bacitracin A and related minor components by liquid chromatography/electrospray ionization ion trap tandem mass spectrometry. Rapid Commun. Mass Spectrom..

[22] Berendsen B.J., Stolker L.A., Nielen M.W. (2013). Selectivity in the sample preparation for the analysis of drug residues in products of animal origin using LC-MS. Trends Anal. Chem..

[23] Kaufmann A., Butcher P., Maden K., Walker S., Widmer M. (2011). Development of an improved high resolution mass spectrometry based multi-residue method for veterinary drugs in various food matrices. Anal. Chim. Acta..

[24] Pradhan A.A., Vera J.H. (1998). Effect of acids and bases on the solubility of amino acids. Fluid Phase Equilibr..

[25] Tseng H.C., Lee C.Y., Wen-Lu Weng W.L., Shiah I.M. (2009). Solubilities of amino acids in water at various pH values under 298.15K. Fluid Phase Equilibr..

[26] Gekko K., Ohmae E., Kameyama K., Takagi T. (1998). Acetonitrile-protein interactions: Amino acid solubility and preferential solvation. Biochim. Biophys. Acta..

[27] Bowden N.A., Sanders J.P.M., Bruins M.E. (2018). Solubility of the Proteinogenic α-Amino Acids in Water, Ethanol, and Ethanol-Water Mixtures. J. Chem. Eng. Data.

[28] Chemicalize. https://chemicalize.com.

[29] Dotsikas Y., Markopoulou C.K., Koundourellis J.E., Loukas Y.L. (2011). Validation of a novel LC-MS/MS method for the quantitation of colistin A and B in human plasma. J. Sep. Sci..

[30] SANCO/2004/2726-rev 4-December 2008 Guidelines for the implementation of Decision 2002/657/EC. https://ec.europa.eu/food/sites/food/files/safety/docs/cs_vet-med-residues_cons_2004-2726rev4_en.pdf.

[31] Song X., Huang Q., Zhang Y., Zhang M., Xie J., He L. (2019). Rapid multiresidue analysis of authorized/banned cyclopolypeptide antibiotics in feed by liquid chromatography-tandem mass spectrometry based on dispersive solid-phase extraction. J. Pharm. Biomed. Anal..

