# Supplementary material for: Determination of Polypeptide Antibiotic Residues in Food of Animal Origin by Ultra-High-Performance Liquid Chromatography-Tandem Mass Spectrometry
Source: Molecules. 2020 Jul 17;25(14):3261. doi: 10.3390/molecules25143261 (PMC7396995; doi:10.3390/molecules25143261)
Supplement: Supplementary file 1 [file molecules-25-03261-s001.pdf]

Supplementary Materials

Determination of polypeptide antibiotic residues in food of animal origin by ultra-high performance liquid chromatography-tandem mass spectrometry

Tomasz Bladek [1], Iwona Szymanek-Bany [1] and Andrzej Posyniak [1]

[1]Department of Pharmacology and Toxicology, National Veterinary Research Institute (NVRI), al. Partyzantów 57, 24-100 Puławy, Poland

Table S1. LC-MS/MS parameters used for monitored compounds.

Analyte	MRM transition (*m/z*)	Declustering potential (V)	Entrance potential (V)	Collision energy (V)	Cell exit potential (V)	Retention time (min)	Ion ratio
Bacitracin A	475.0 → 199.0[a]	50	10	35	26	2.12	1.75
	475.0 → 227.0	50	10	28	10		
Colistin A	390.7 → 384.6[a]	50	10	16	15	1.80	1.08
	390.7 → 101.1	50	10	23	15		
Colistin B	386.0 → 380.0[a]	50	10	16	15	1.70	1.21
	386.0 → 101.1	50	10	23	15		
Polymyxin B1	402.0 → 396.0[a]	50	10	16	16	1.84	1.22
	402.0 → 101.1	50	10	24	15		
Polymyxin B2	397.5 → 391.4[a]	50	10	15	15	1.75	1.47
	397.5 → 101.1	50	10	25	15		

[a] ion transition used for quantification

Table S2. Some physico-chemical properties of studied compounds [28].

Analyte	Strongest acidic pKa	Strongest basic pKa	Isoelectric point (pI)	octanol/water partition coefficient (logP)
Bacitracin A	3.74	13.46	8.06	-6.89
Colistin A	9.07	10.24	10.41	-7.65
Colistin B	9.07	10.24	10.41	-8.10
Polymyxin B1	9.07	10.24	10.42	-7.25
Polymyxin B2	9.07	10.24	10.42	-7.69

Table S3. Variables and their levels in the Youden ruggedness test experimental design.

Selected variables	Units	Abbreviation[a]	High level	Low level
Amount of 25% ammonia	ml[b]	A, a	10.2	9.8
Amount of water	ml[b]	B, b	10.2	9.8
Volume of extraction solution	ml	C, c	8.2	7.8
Time of shaking	ml	D, d	11	9
Time of sonication	min	E, e	11	9
Time of centrifugation	min	F, f	11	9
Temperature of evaporation	°C	G, g	46	44

[a]Upper case letter represents high level, lower case letter represents low level value of the quantitative variable; [b]The changes were designed considering the preparation of 100 mL of extraction mixture

Table S4. Statistical evaluation of ruggedness test results (7 factors, 8 experiments) for muscle samples.

Selected variables	Bacitracin A	Colistin A	Colistin B	Polymyxin B1	Polymyxin B2
SD_{WLR}[a]	12.2	14.7	13.4	14.1	13.5
SD_i[b]	9.80	10.0	10.5	10.1	9.10
t_{crit}[c]			2.11		
	t[d]	t[d]	t[d]	t[d]	t[d]
Amount of 25% ammonia	1.42	1.27	1.37	1.13	1.18
Amount of water	0.95	0.79	1.16	1.08	0.86
Volume of extraction solution	0.61	0.46	0.37	0.38	0.18
Time of shaking	0.72	0.60	0.68	0.68	0.65
Time of sonication	0.43	0.41	0.42	0.43	0.44
Time of centrifugation	0.14	0.12	0.21	0.18	0.29
Temperature of evaporation	0.66	0.50	0.53	0.58	0.55

[a]SD_{WLR} – standard deviation of within-laboratory reproducibility; [b]SD_i – standard deviation of differences; [c]t_{crit} – critical value of *t*-test (95%); [d]*t* – experimental value of the *t*-test

Table S5. Preparation of the calibration curves and the spiked samples.

Analyte spiking level (μg kg^{-1})	Concentration of working standard solution (μg mL^{-1})	Added volume of working standard solution (μL)	Final volume (mL)	Final concentration of analyte in calibration curve (ng mL^{-1})
10[a]	2	10	1	20
25[b]	2	25	1	50
50[c]	2	50	1	100
75[bd]	2	75	1	150
100[e]	2	100	1	200
150[def]	2	150	1	300
225[d]	2	225	1	450
300[f]	20	30	1	600
450[f]	20	45	1	900
1000[a]	20	100	1	2000

[a]Level selected to all analytes in all tissue; [b]level selected for colistin A and B and polymyxin B1 and B2 in milk; [c]level selected for all analytes in milk; [d]level selected for all analytes in muscle; [e]level selected for bacitracin A in milk; [f]level selected for all analytes in eggs
